# Kidney stone detection via axial CT imaging: A dataset for AI and deep learning applications

**DOI:** 10.1016/j.dib.2025.111446

**Published:** 2025-03-05

**Authors:** Peshraw Ahmed Abdalla, Muhammad Y. Shakor, Aso Khaleel Ameen, Bander Sidiq Mahmood, Nawzad Rasul Hama

**Affiliations:** a Computer Science Department, College of Science, University of Halabja, Kurdistan Region, Halabja 46018, Iraq; b Information Technology Department, College of Computer and Information Technology, University of Garmian, Kalar 46021, Iraq; c Department of Computer Science, College of Science, Knowledge University, Erbil 44001, Iraq; d Department of Information System, ICTC Directorate, Ministry of High Education and Scientific Research, Kurdistan Region, F.R, Iraq

**Keywords:** Medical dataset, Clinical AI, Axial CT image dataset, Kidney stone detection, Deep learning, Artificial intelligence, Image augmentation

## Abstract

This article introduces a comprehensive CT scan image dataset focused on kidney stone detection, consisting of two groups: one drawn from patients with kidney stones and the other from patients without kidney stones. This dataset has been cleaned, cross-checked, and checked adequately before labeling in coordination with the medical experts from the medical field. Samples in the dataset were derived from different health facilities in Sulaimani and Rania, Iraq, which supplied crucial information about the demographics and patterns of kidney stones in the area. It holds 3364 original CT images and 35,457 augmented CT images, which can be used to create deep-learning models for kidney stone diagnosis. The enhanced images also make it possible to use them in training or developing medical practice and educational algorithms. This dataset can be an asset in developing new diagnostic tools, supporting medical research, and being used as learning material for students studying in the medical field.

Specifications Table

**Table:** 

Subject	Computer Vision, Pattern Recognition, Artificial Intelligence
Specific subject area	Machine Learning; Kidney Stone Detection; Image Processing
Type of data	Image. Raw, Cleaned Data, Annotated Data, Augmented Data
Data collection	The dataset includes 3364 primary CT images with a 1920 × 1080 pixels resolution sourced from the medical centers of Sulaimani and Rania, Kurdistan Region, Iraq. The images were taken using kidney CT scanners, and they were checked and annotated by professional radiologists and medical workers to achieve high image quality. Additionally, the dataset is divided into two categories: stone and non-stone. It is a balanced and reliable resource for research and academic purposes.
Data source location	Institution1: Faruq Medical City City/Town/Region: Country: Sulaimani City, Kurdistan Region Institution2: Rania Hospital City/Town/Region: Rania, Sulaimani City, Kurdistan Region Country: Iraq Institution3: Mercy Medical City City/Town/Region: Sulaimani City, Kurdistan Region Country: Iraq
Data accessibility	Repository name: Mendeley Data Data identification number: 10.17632/fwhytt5mzd.2 Direct URL to data: Axial CT Imaging Dataset for AI-Powered Kidney Stone Detection: A Resource for Deep Learning Research - Mendeley Data [1].
Related research article	None

## 1 Value of the Data

• **Advances Medical Imaging Research:** The dataset provides a foundational resource for researchers developing and improving algorithms for image segmentation, feature extraction, and classification. It thereby supports innovation for clinical AI, which can facilitate the processing and analysis of CT scans.
• **Supports Kidney Stone Detection Model Development:** This dataset offers an extensive, annotated collection of CT images, essential for training machine learning models for automatic kidney stone detection, enhancing the accuracy and efficiency of diagnostic tools in clinical practice.
• **Encourages Cross-Institutional Collaboration:** Including data from multiple hospitals ensures diversity in patient demographics and imaging systems, promoting robust models that generalize across varied clinical environments while fostering collaboration among institutions.
• **Provides an Educational and Training Resource:** The dataset is a practical aid for healthcare and industrial training in kidney stone detection, medical imaging, and machine learning and provides fundamental teaching and benchmarking for the applications of new algorithms.
• **Standardized and Accessible for Reuse:** The dataset is augmented with the JPG format and the uniform 512 × 512 resolution, which makes it interoperable with various machine learning frameworks. This makes it a perfect solution for a wide range of medical imaging innovations and developments due to the ease of using it repeatedly.
• **Ensures High-Quality and Reliable Annotations:** The dataset is verified by expert radiologists and is equipped with labels that guarantee high-quality annotations, a prerequisite for reproducibility and reliability in research and educational applications.

## 2 Background

The creation of this dataset was initiated due to the fact that the medical sector will have more and more automated diagnostic tools [2]. The applications of AI in the medical field are accelerating. In particular, kidney stone detection is on the rise. Kidney stones are a widespread disease, but they are life-threatening and need timely treatment in case of an emergency. To treat and detect this disease, doctors use conventional CT scans and radiologists’ experience, which is a time-consuming process since they derive it from their expertise [3,4]. Although radiologists can cause a discrepancy in the interpretation of diagnostic imaging techniques, a significant number of pathologies identified by AI are not identified by a skilled radiologist. Thus, the dataset represents a significant clinical and public health issue.

This dataset has been constructed with the goal of using AI to detect kidney stones from CT scans, and it's in the process of trying to improve those methods. That is, it has varied and numerous collections of labeled CT images that have been obtained from several hospitals to train the machine learning models. The variety of the dataset assures the refinement of algorithms that have the capability to identify kidney stones under different imaging conditions and across various patient demographics. Also, this dataset is distinguished by its extensive diversity, substantial size, and application of systematic image augmentation techniques.

This dataset provides a standardized collection of real-world cases intended for training and validating machine learning algorithms, aiding the development of efficient and accurate diagnostic tools in healthcare.

## 3 Data Description

The dataset showcases kidney stone detection by leveraging high-resolution CT scan images. It comprises 3364 original CT images and 35,457 augmented images collected from different hospitals and medical centers. They represent various patient populations and imaging modalities. Two classes organize the images, stone and non-stone, and the annotations are provided at the most detailed level to help nephrologists, radiologists, and AI researchers. Original images are in the JPG format with a resolution of 1920 × 1080 pixels, while the augmented images are resized to 512 × 512 pixels for computational efficiency. Each CT scan is segmented into three anatomical planes—coronal, axial, and sagittal—providing comprehensive coverage of kidney anatomy.

**Fig. 1:**
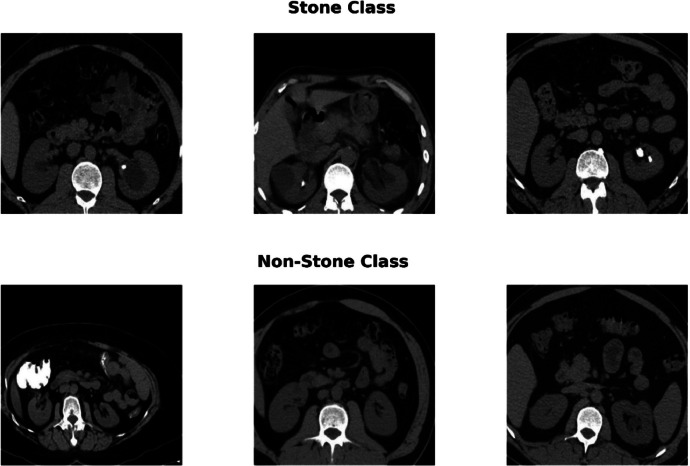
Representative samples of kidney CT images: stone and non-stone classes.

Fig. 1 shows samples from the kidney stone dataset that are typical for the two main categories of Stone and Non-Stone. Stone class samples are shown in the upper row, and non-stone class samples in the lower row. These images have been carefully curated to depict key features for both categories, which are vital for training machine learning models to automate kidney stone detection.

### 3.1 Data sources and collection methods

The CT imaging sources used to generate the research dataset were devices acquired from three medical centers: Mercy Medical City, Faruq Medical City, and Rania Hospital, as illustrated in Table 1. The scans were collected between June 2024 and September 2024, thereby ensuring the representation of the patient demographic profile. Modern CT scanners with standardized protocols complete all the imaging protocols to maintain the coherence of the image quality.

**Table 1:** Distribution of patients, images, and classifications across medical centers.

Hospital/Medical Center	Number of Patients	Male	Female	Number of Images	Stone	Non-stone
**Mercy Medical City**	29	17	12	938	461	477
**Faruq Medical City**	65	43	22	448	146	302
**Rania Hospital**	107	60	47	1978	970	1008
**Total**	201	120	81	3364	1577	1787

The dataset comprises images acquired using advanced CT imaging systems, ensuring high consistency and quality. Each scan was segmented into three distinct planes such as:

• Coronal views offer a longitudinal perspective of the kidney.
• Axial views provide cross-sectional slices for detailed structural analysis.
• Sagittal views highlight the kidney's lateral dimensions.

This multi-plane observational approach enables robust AI model training by incorporating diverse physiological parameters of the kidney. For this study, axial views were selected for analysis. The dataset includes scans from individuals aged 20 to 65, with an approximately balanced gender distribution. Data labelling was conducted by experienced radiologists, ensuring high reliability and accuracy in annotations.

**Fig. 2:**
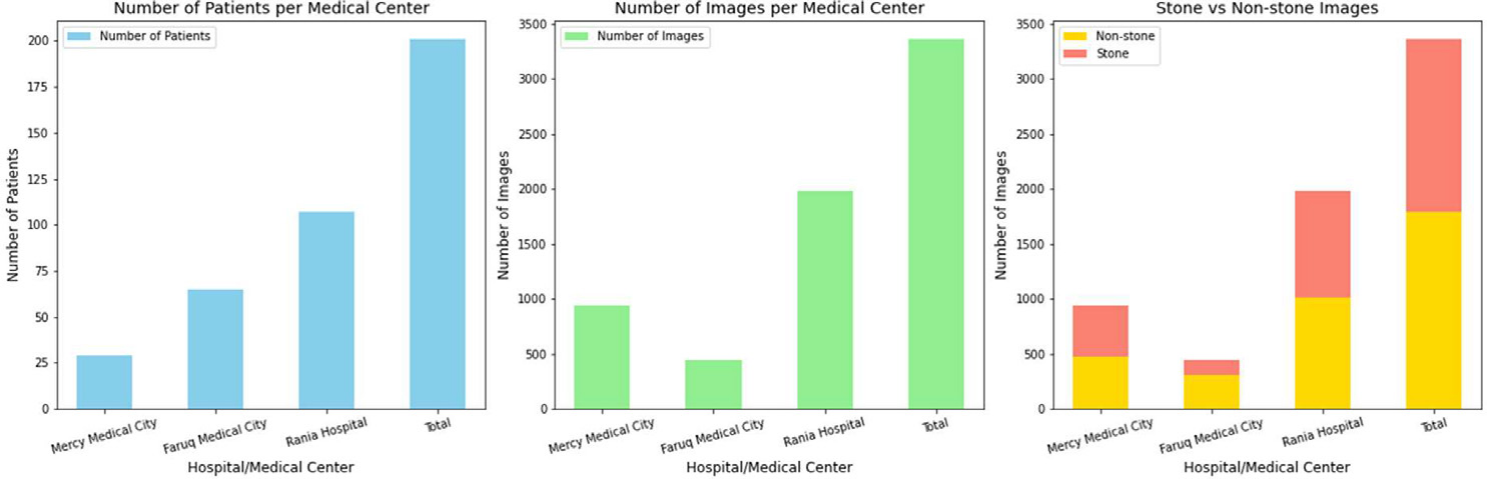
Distribution of patients, images, and stone classifications across medical centers.

Fig. 2 provides a comprehensive visualization of the dataset collected from three medical centers, highlighting the distribution of patients, total images, and their classifications. The first subplot illustrates the **number of patients** treated at each center, with Rania Hospital having the highest patient count (107) and Mercy Medical City having the lowest (29).

The second subplot showcases the image distribution received from different healthcare facilities. Rania Hospital presented the biggest number of pictures, almost 1978, and Faruq Medical City provided the lowest, 448 images. By the way, Mercy Medical City, a medical center with fewer patients, added 938 images.

The third subplot provide an expanded view on the classifications providing a stacked bar chart with stone and non-stone classes. Rania Hospital performed the highest numbers of both stone (970) and non-stone (1008) images. In contrast Faruq Medical City 146 of the images were stone and 302 of the images non-stone. Mercy Medical City made a fairly even mixture where 461 stones and 477 non-stone images were captured. All these visualizations give a comparative analysis of the contributions of data and distribution of classes across the medical centers.

## 4 Experimental Design, Materials and Methods

### 4.1 Data collection

This work complied with relevant ethical approval and permissions from all the hospitals involved; the actual approval and permission were obtained from the institutions involved in the research. Kidney CT scan images were collected using modern imaging tools, and the study was coordinated by the nephrology and radiology staff. The aim was to get images that allowed for the distinction of kidney stones and maximize the benefits of CT over potentially useful techniques like MRI or ultrasound.

The data used in this study was collected from several healthcare centers obtained from various locations, such as Rania Hospital, Mercy Medical City, and Farouk Medical City. For the purpose of diversity, the images were gathered from patients of both genders and a variety of ages. Although there were difficulties in attracting all the institutions planned for participation in the project, the obtained set of images presents a diverse range of images. This monograph provides the basis for developing enhanced automated diagnosis tools for kidney stone detection.

### 4.2 Data augmentation

To expand the dataset and improve the adaptability of deep-learning models in medical image analysis, a variety of image augmentations were used. These techniques are essential for expanding the training data set so that the model can handle clinical diversity in the real world and for enhancing the model's capacity for generalization [5]. A Python script utilizing the *imgaug* library was employed, implementing twelve sequential augmentation techniques to introduce variations and distortions [6]. The following strategies were applied:

1. **Rotation**: Randomly rotates images within a range of −45° to 45°, altering their orientation.
2. **Piecewise Affine Transformation**: This method applies a piecewise affine transformation with a random scale (0.02–0.1), locally distorting the image.
3. **Dropout**: Randomly sets a fraction (0–0.2) of image pixels to zero, simulating missing or corrupted data.
4. **Perspective Transformation:** Adjusts the perspective of images within a transformation range of 0.05–0.15, simulating changes in viewing angles.
5. **Linear Contrast**: Alters image contrast by a random factor between 0.2 and 3.0, adjusting the pixel intensity distribution.
6. **Multiplication**: Modifies image brightness by multiplying pixel values with a random factor between 0.5 and 1.5.
7. **Flipping (Horizontal)**: Horizontally flips images with a probability of 1.
8. **Flipping (Vertical)**: Vertically flips images with a probability of 1, creating a mirror effect along the vertical axis.
9. **Gaussian Blur**: Applies Gaussian blur with random sigma values between 0 and 2.0 to smooth images, reduce noise, and enhance edges.
10. **Gaussian Noise:** Introduces Gaussian noise with a random scale of 0–0.2 times 255, mimicking real-world image noise.
11. **Cropping**: Randomly crops images by a percentage (0–0.2), removing parts of the image and focusing on specific areas of interest.
12. **Elastic Transformation**: Applies elastic transformations with a random alpha value between 0 and 10.0, simulating deformations in the image.

**Fig. 3:**
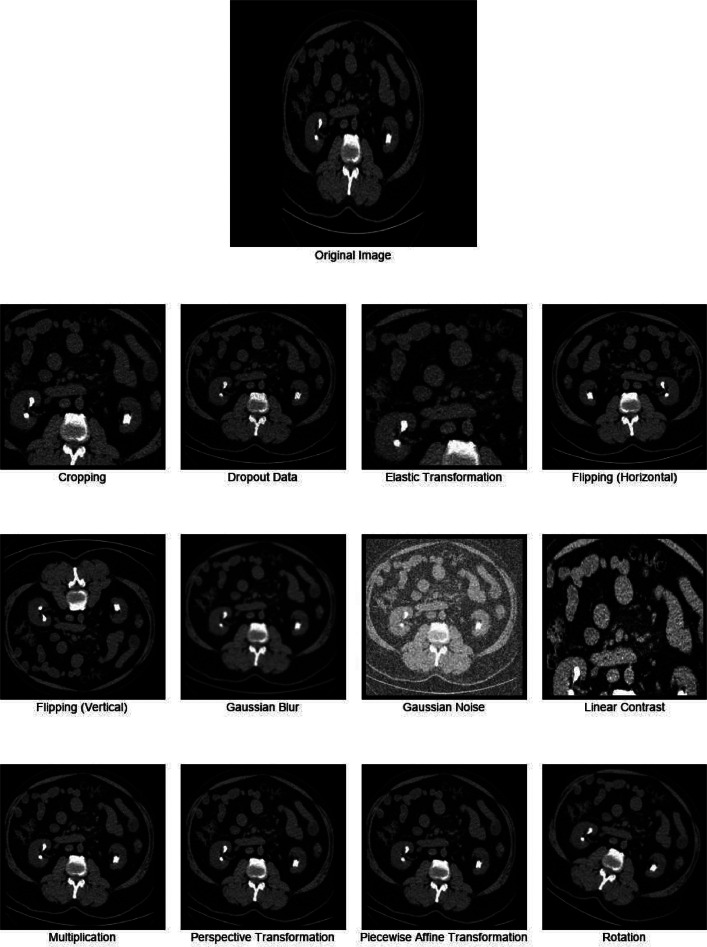
Examples of applying augmentation techniques to enhance dataset diversity for model training.

Fig. 3 demonstrates the original medical image alongside its augmented variants using various data augmentation techniques, such as cropping, flipping, and Gaussian noise addition.

The original CT scan dataset, which consisted of 3364 images, was resized to 512 × 512 pixels. These augmentation methods were utilized to increase data diversity and improve the robustness of the model during training. As a result of the augmentation process, the dataset expanded to 35,457 images, excluding the original images. However, certain augmentation techniques yielded fewer images due to the exclusion of scans with poor kidney visibility, corrupted structures, or cases where only a partial view of the kidney was available. This quality control measure ensured that the dataset maintained its integrity, with high-quality images selected for model training. A detailed summary of the applied augmentation techniques and the resulting number of images is presented in Table 2.

**Table 2:** Summary table of augmentation techniques.

Augmentation Technique	Images Generated	Corrupted Images Removed	Final Images	Images with Stone	Images without Stone	Cumulative Total
Cropping	3364	1982	1382	522	860	1382
Dropout Data	3364	0	3364	1577	1787	4746
Elastic Transformation	3364	758	2606	1251	1355	7352
Flipping (Horizontal)	3364	0	3364	1577	1787	10716
Flipping (Vertical)	3364	0	3364	1577	1787	14080
Gaussian Blur	3364	0	3364	1577	1787	17444
Gaussian Noise	3364	298	3066	1279	1787	20510
Linear Contrast	3364	1770	1594	602	992	22104
Multiplication	3364	103	3261	1524	1737	25365
Perspective Transformation	3364	0	3364	1577	1787	28729
Piecewise Affine Transformation	3364	0	3364	1577	1787	32093
Rotation	3364	0	3364	1577	1787	35457
**Total**	**40368**	**4911**	**35457**	**16217**	**19240**	**35457**

**Fig. 4:**
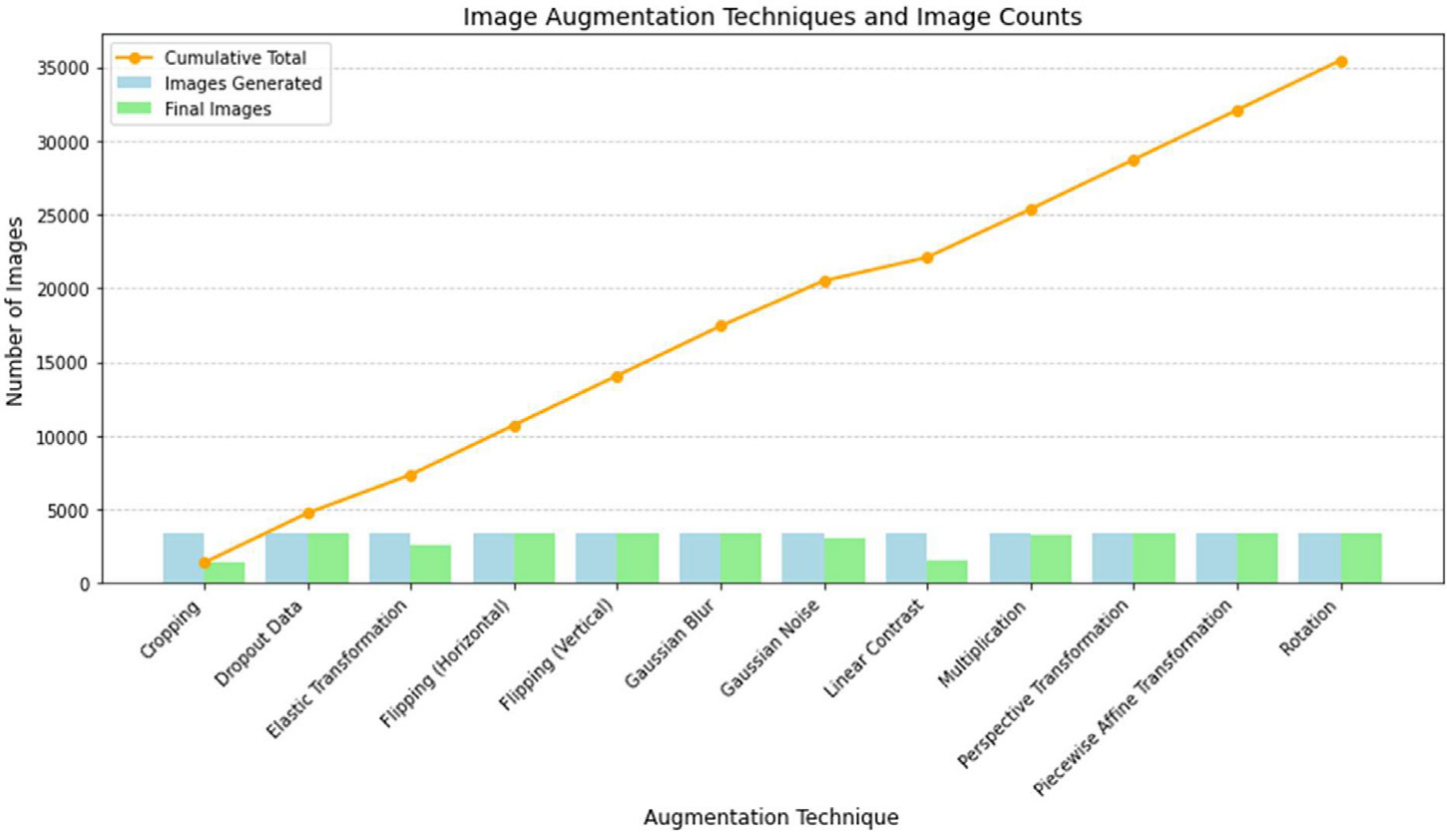
Distribution of generated and final images across various augmentation techniques.

Fig. 4 illustrates the generated and final image distribution for each augmentation technique, with clear visual distinction between the two. The bars for **Images Generated** (light blue) are shifted slightly to the left, and the bars for **Final Images** (light green) are shifted to the right, preventing overlap and allowing for a side-by-side comparison. Additionally, the **Cumulative Total** (orange line) tracks the total number of images across the techniques, highlighting the growing impact of each augmentation method.

**Fig. 5:**
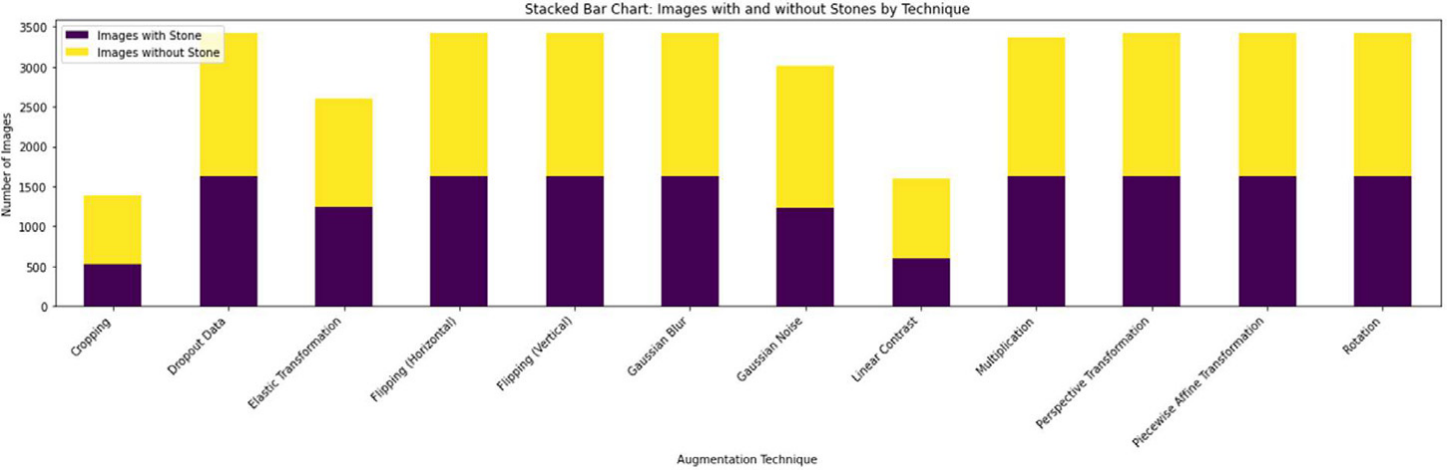
Distribution of images with and without stones across the applied image augmentation techniques.

The chart (Fig. 5) is a stacked bar visualization showing the distribution of images with and without stones for each augmentation technique. The purple bars represent images with stones, while the yellow bars show images without stones. This comparison reveals the effect of each technique on the balance between these two categories.

**Fig. 6:**
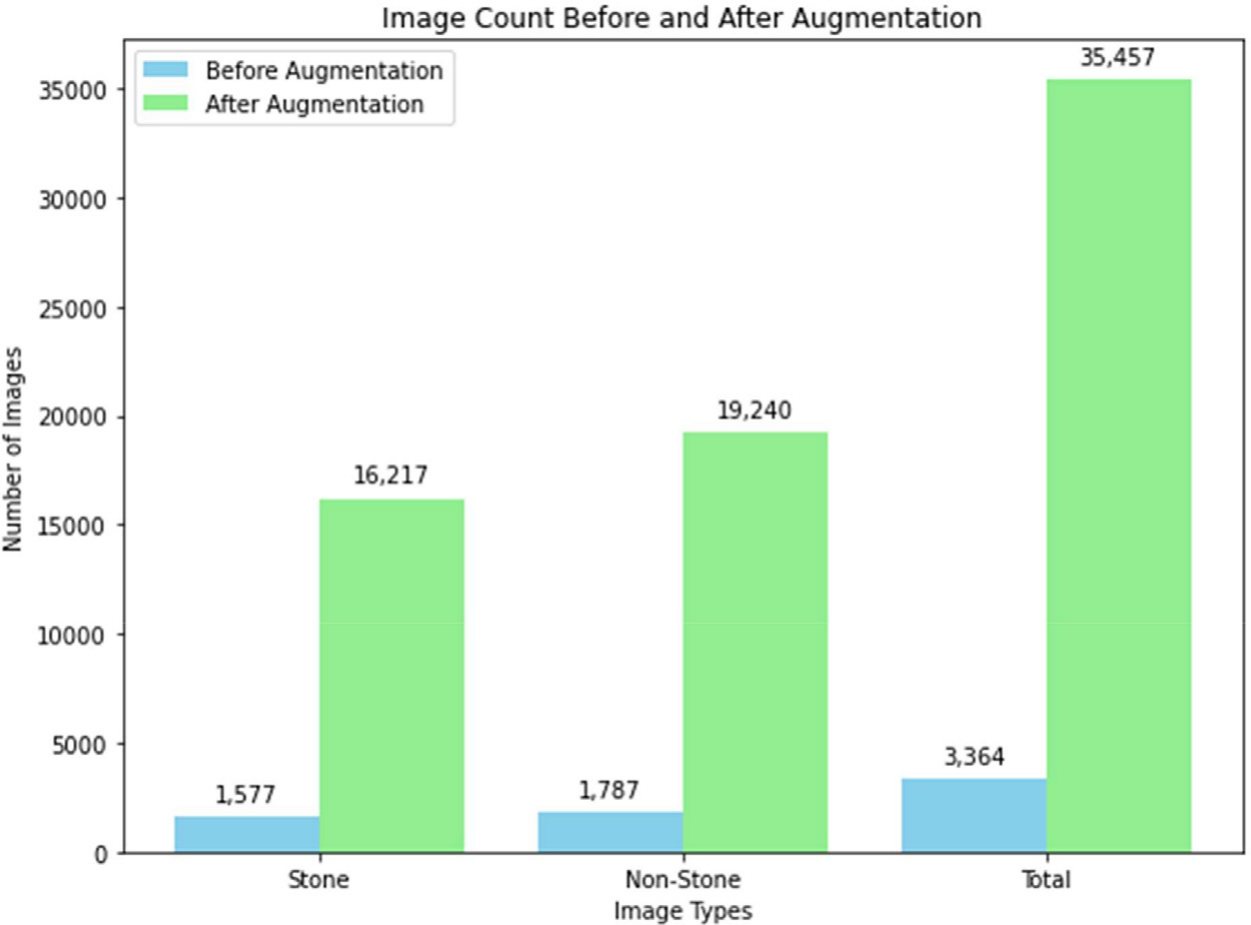
Comparison of image counts before and after augmentation across stone, non-stone, and total categories.

Fig. 6 compares the number of images before and after applying augmentation techniques across three categories: ``Stone,'' ``Non-Stone,'' and ``Total.'' Initially, there were **1577 stone images** and **1787 non-stone images**, summing to **3364 total images**. Following augmentation, the image count increased significantly to **16,217 stone images** and **19,240 non-stone images**, resulting in a total of **35,457 images**. The augmentation process amplified the dataset to improve model performance and generalization capabilities.

**Fig. 7:**
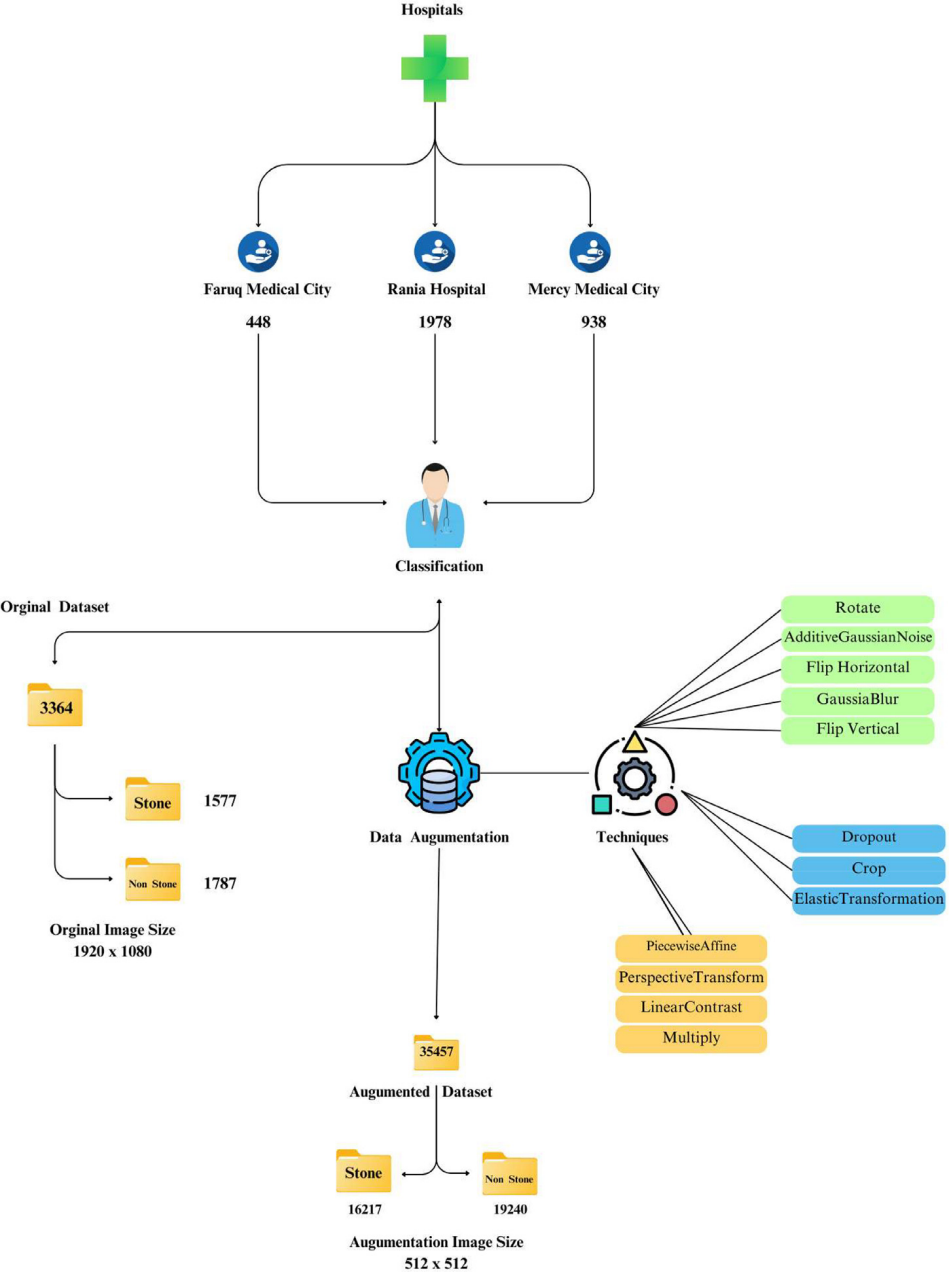
Workflow of medical image classification with data augmentation.

Fig. 7 illustrates the medical image classification process, where the original dataset of 3364 images (1920 × 1080 resolution) from three hospitals is augmented using techniques like rotation and flipping. This increases the dataset to 35,457 images, each resized to 512 × 512, enhancing model training.

### 4.3 Labelling strategy

To ensure a well-structured and reliable dataset, we have implemented a systematic labelling strategy that uniquely identifies each image while maintaining patient privacy. The dataset follows a structured labelling format to ensure clear identification of each image, with components such as gender, stone status, patient ID, image number, hospital name, and augmentation technique as explained in Table 3. This standardized approach facilitates dataset organization and enhances reproducibility in research.

**Table 3:** Labelling components and descriptions.

Label	Abbreviation	Description
**Patient Number**	PXXX	Unique patient identifier (e.g., P037 means patient 37)
**Hospital Name**	FA	Faruq Hospital
	ME	Mercy Hospital
	RA	Rania Hospital
**Gender**	M	Male
	F	Female
**Stone Status**	S	Stone
	NS	Non-Stone
**Image Count**	IXX	Number of images for that patient (e.g., I01 means image 1)
**Augmentation Techniques**	CR	Cropping
	DO	Dropout
	EL	Elastic transformation
	FH	Horizontal flip
	FV	Vertical flip
	GB	Gaussian blur
	GN	Gaussian noise
	LC	Linear contrast
	MUL	Multiplication
	PE	Perspective transformation
	PA	Piecewise affine
	ROT	Rotation

This strategy enables the application of a patient-wise data split, ensuring that all images from a single patient are assigned exclusively to one dataset partition (training, validation, or testing). This prevents data leakage and eliminates the risk of overestimated performance caused by overlapping images across different dataset splits.

### 4.4 Labelling format

Each image in the dataset is assigned a structured label that encodes essential metadata. The labelling conventions for both original and augmented images are defined as follows:

**Original Image Format:** PatientID_HospitalName_Gender_StoneStatus_ImageNumber

**Augmented Images:** PatientID_HospitalName_Gender_StoneStatus_ImageNumber_TechniqueName

For example, an original image is labelled as **P189_RA_F_S_I10**, where ``P189'' represents the unique patient ID, ``RA'' refers to the hospital where the scan was obtained which is Rania, ``F'' indicates a female patient, ``S'' denotes the presence of a kidney stone, and ``I10'' is the 10th image for the patient ID of ``P189''. In the case of an augmented image, the label follows the same structure with an additional suffix indicating the applied augmentation technique. For instance, **P189_RA_F_S_I10_EL** represents the same original image after undergoing an elastic transformation. This structured labelling ensures clear identification and facilitates proper dataset organization.

## Limitations

• **Poor Image Quality:** Some CT images, particularly from patients under the age of sixteen, were of low quality, blurry, or unclear. These images were excluded to maintain consistency and quality for model training.
• **Other Pre-existing Conditions:** Images from patients with severe conditions, such as tumors or kidney-related complications, were removed as these conditions made kidney stone identification unclear, avoiding confusion in the detection process.
• **Corrupted Images:** A few CT images were corrupted or lacked essential data, rendering them unusable for analysis. These images were excluded from the dataset.
• **Small Image Size:** Certain CT images had small dimensions, making it difficult to extract sufficient details. These images were excluded due to insufficient resolution for proper feature extraction.
• **Incomplete Labels:** Some images had incomplete labelling, where only one kidney stone was detected, leading to inconsistencies. These images were excluded to ensure accurate and complete data.
• **Hospital Collaboration Issues:** Some hospitals were unable to provide additional CT scan data, limiting the dataset's size and diversity.

## Ethics Statement

Researchers conducting this study recognize the critical importance of protecting patient privacy and maintaining the confidentiality of health information. All patients and hospitals involved were fully informed about the study's objectives and the intended use of their medical data. To ensure privacy, the data collected for this research were anonymized, and no identifiable information was retained or disclosed at any stage of the study.

Informed consent was obtained from all participants and relevant parties, including explicit permission from patients to use their anonymized medical images for research purposes. Patient confidentiality was rigorously upheld throughout the research process in accordance with established ethical standards.

The authors confirm that they have read and follow the ethical requirements for publication in Data in Brief. This work does not involve direct human subjects, animal experiments, or data collected from social media platforms.

Ethical committee approval was not required for this study, as the research did not involve direct interaction with human participants or experiments. The dataset complied fully with ethical guidelines and institutional policies regarding data management and research practices.

The authors ensured that the research was conducted responsibly, transparently, and in compliance with all relevant ethical principles.

## CRediT authorship contribution statement

**Peshraw Ahmed Abdalla:** Writing – review & editing, Supervision, Methodology, Software, Project administration. **Muhammad Y. Shakor:** Writing – review & editing, Data curation. **Aso Khaleel Ameen:** Writing – review & editing. **Bander Sidiq Mahmood:** Writing – original draft, Data curation, Resources, Software. **Nawzad Rasul Hama:** Writing – original draft, Data curation, Resources, Software.

## Data Availability

Mendeley DataAxial CT Imaging Dataset for AI-Powered Kidney Stone Detection: A Resource for Deep Learning Research (Original data).
